# Effectiveness of Motor Imagery on Motor Recovery in Patients with Multiple Sclerosis: Systematic Review

**DOI:** 10.3390/ijerph18020498

**Published:** 2021-01-09

**Authors:** Ana Gil-Bermejo-Bernardez-Zerpa, Jose A. Moral-Munoz, David Lucena-Anton, Carlos Luque-Moreno

**Affiliations:** 1 Department of Nursing and Physiotherapy, University of Cadiz, 11009 Cadiz, Spain; ana.gilbermejober@alum.uca.es (A.G.-B.-B.-Z.); joseantonio.moral@uca.es (J.A.M.-M.); carlos.luque@uca.es (C.L.-M.); 2 Institute of Research and Innovation in Biomedical Sciences of the Province of Cadiz (INiBICA), University of Cadiz, 11009 Cadiz, Spain

**Keywords:** motor imagery, mental processes, psychomotor performance, rehabilitation, multiple sclerosis

## Abstract

The effects of motor imagery (MI) on functional recovery of patients with neurological pathologies, such as stroke, has been recently proven. The aim of this study is to evaluate the effectiveness of MI on motor recovery and quality of life (QOL) in patients with multiple sclerosis (pwMS). A search was carried out in the following scientific databases: PubMed, CINAHL, PEDro, Scopus, Cochrane and Web of Science, up to November 2020. The grey literature and reference lists of potentially relevant articles were also searched. The Checklist for Measuring Quality and The Cochrane collaboration’s tool were used to assess the methodological quality and risk of bias of the studies. Five studies were included in the systematic review. Findings showed that pwMS using MI had significant improvements in walking speed and distance, fatigue and QOL. In addition, several benefits were also found in dynamic balance and perceived walking ability. Although the evidence is limited, rehabilitation using MI with the application of musical and verbal guides (compared to non-intervention or other interventions), can produce benefits on gait, fatigue and QOL in pwMS with a low score in the Expanded Disability Status Scale.

## 1. Introduction

Multiple sclerosis is a demyelinating, chronic and autoimmune neurological pathology. Morphologically, it presents with inflammation, demyelination and axonal damage, which affects the entire central nervous system, causing very varied symptoms [1]; thus, it represents the most common chronic neurological disease in young adults [2]. The quality of life (QOL) and functional outcomes of patients with multiple sclerosis (pwMS), such as mobility, aerobic performance and muscle strength, can be improved through the implementation of structured and multidisciplinary rehabilitation programmes and physical therapy [3]. In addition to conventional physical therapy, there are many interventions focused on complement these programmes [4]. Some methods, such as virtual reality, count with already proven evidence in pwMS [5]. In this way, the applicability of motor imagery (MI) techniques has recently been shown in different rehabilitation contexts [6]. MI is defined as a mental practice of movements without actually executing them. Parts of the cortical networks involved in motor control that are activated during actual movement are also activated during MI [7]. MI is widely used for the study of cognitive aspects of action control and can be applied in various motor training and (re)learning environments, both in the healthy population and clinical conditions [8,9]. In 1964, Richardson first discussed the possibility of using mental practise through MI as a practical technique for physical therapists in the motor rehabilitation process [10].

Recently, several review articles analysed MI interventions in various neurological disorders (stroke, Parkinson’s disease, spinal cord injury and amputation), underscoring the positive effects of mental training on motor performance [11,12,13] and highlighting changes in motor impairment favoured by MI [14,15]. Specifically, promising findings were reported on gait and fatigue improvement in pwMS [16]. Although some reviews provided relevant information on this topic [17], some of them did not specifically focus on pwMS or did not exclusively consider MI as a form of intervention [18]. Moreover, no systematic review has been reported on MI effectiveness in pwMS that specifically focused on functional and motor recovery. Relevant here, it would be of interest take into account the effects produced by other forms of mental practice on variables as important as pain [19]. Recent studies support the interest of clinicians in obtaining evidence on the effectiveness of this technique [20,21] as well as in knowing the most important parameters that optimise its application. Considering the extensive data available on MI as a treatment option in neurological disorders other than multiple sclerosis [13,22,23], it is important to gather data on MI as a tool to be used by physical therapists dealing with pwMS. For this reason, the main objective of this systematic review was to analyse the effectiveness of the MI technique in the rehabilitation of pwMS, first obtaining a general view of literature on this issue, then summarising benefits and limitations of MI, and finally evaluating and comparing the most studied variables (gait, balance, fatigue and QOL) relevant to the effectiveness of MI in treatment of pwMS. 

## 2. Materials and Methods

This study followed the scheme of a systematic review concerning the Preferred Reporting Items for Systematic Review and Meta-Analysis (PRISMA) model [24].

### 2.1. Search Strategy

An exhaustive search was performed up to November 2020 in the most common scientific databases: Medline/PubMed, Scopus, Cochrane Plus, CINAHL, Web of Science and PEDro. In addition, a Google Scholar database search was conducted to find ‘grey’ literature and the reference lists of retrieved papers were examined to identify further relevant studies. The following descriptors obtained from MeSH were used to build the search query: “multiple sclerosis”, “rehabilitation”. Other free terms, “motor imagery”, “mental imagery” and “mental practice”, were also used due to the absence of specific MeSH descriptors. The detailed search strategy for PubMed database is shown in Supplementary Materials (Table S1). No filters related to the date of publication were used. The results were filtered to obtain studies published in English, Spanish and French languages. Search strategies in the different databases are shown in Table 1.

### 2.2. Eligibility Criteria

The articles were selected based on the research question, elaborated following the PICOS model [25]: (1) Population: adults pwMS, (2) Intervention: interventions using MI isolated (non-combined) involving any internal/external stimulus, such as auditory guides, visual guides, among others; (3) Comparison: conventional physical therapy, no intervention, and other therapies; (4) Outcomes: motor improvements, QOL and fatigue; and (5) Study type: clinical trials (controlled and non-controlled, randomised and non-randomised) and pilot studies. Only the results related to pwMS were considered when the studies included data about other neurological pathologies. 

### 2.3. Assessment of the Methodological Quality and Risk of Bias

The Checklist for Measuring Study Quality [26] was used to analyse the methodological quality of the included studies. This tool uses 27 questions (“yes”, “no” or “unable to determine”) through five different sections related to the overall quality of the study, the ability to generalise findings of the study, the assessment of the bias in the intervention and outcome measure(s), the assessment of the bias from sampling or group assignment, and the possibility that findings are due to chance. 

The assessment of the risk of bias was performed through The Cochrane Collaboration’s tool [27], using the Review Manager 5.4 software (the Cochrane Collaboration, The Nordic Cochrane Centre, Copenhagen, Denmark). This tool allows the evaluation and rating of the risk of bias in terms of “low risk”, “high risk” and “unclear risk”.

### 2.4. Selection Process and Data Extraction

The search was conducted using the different keywords previously described in the different scientific databases. After reading in detail the title and abstract, according to the previous criteria, potentially relevant articles were identified, and the duplicated documents were excluded. Finally, an exhaustive verification of the inclusion criteria applied to the articles included in this systematic review was carried out. 

Two independent reviewers (A.G.-B.-B.-Z. and C.L.-M.) were actively involved in study selection, review, and systematic data extraction from each included study. An additional reviewer (D.L.-A.) took part in the consensus of the different decisions. The following information was extracted from each article: author, year of publication, demographic characteristics of the participants (total number of participants, number of participants in both groups, average age), as well as the functional stage of participants, and characteristics of the intervention carried out (the type of MI, intensity and timing of the sessions, measurement instruments, and results).

## 3. Results

**Figure 1:**
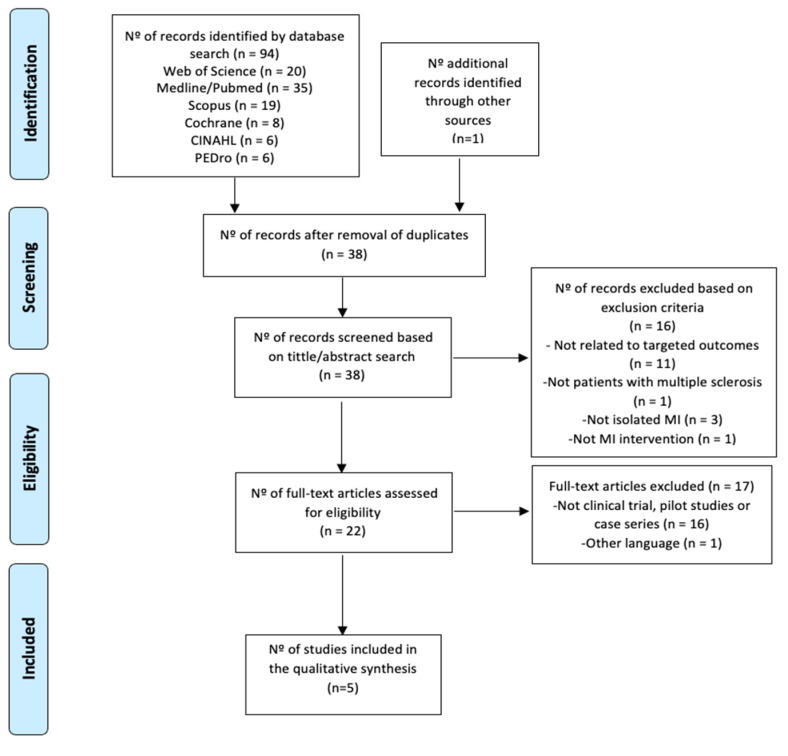
Information flowchart of the different phases of the systematic review.

From a total of 70 studies initially identified, only five were considered for a qualitative synthesis (Figure 1). A list of the excluded articles and reasons, is shown in Supplementary Materials (List S1).

### 3.1. Methodological Quality and Risk of Bias

**Figure 2:**
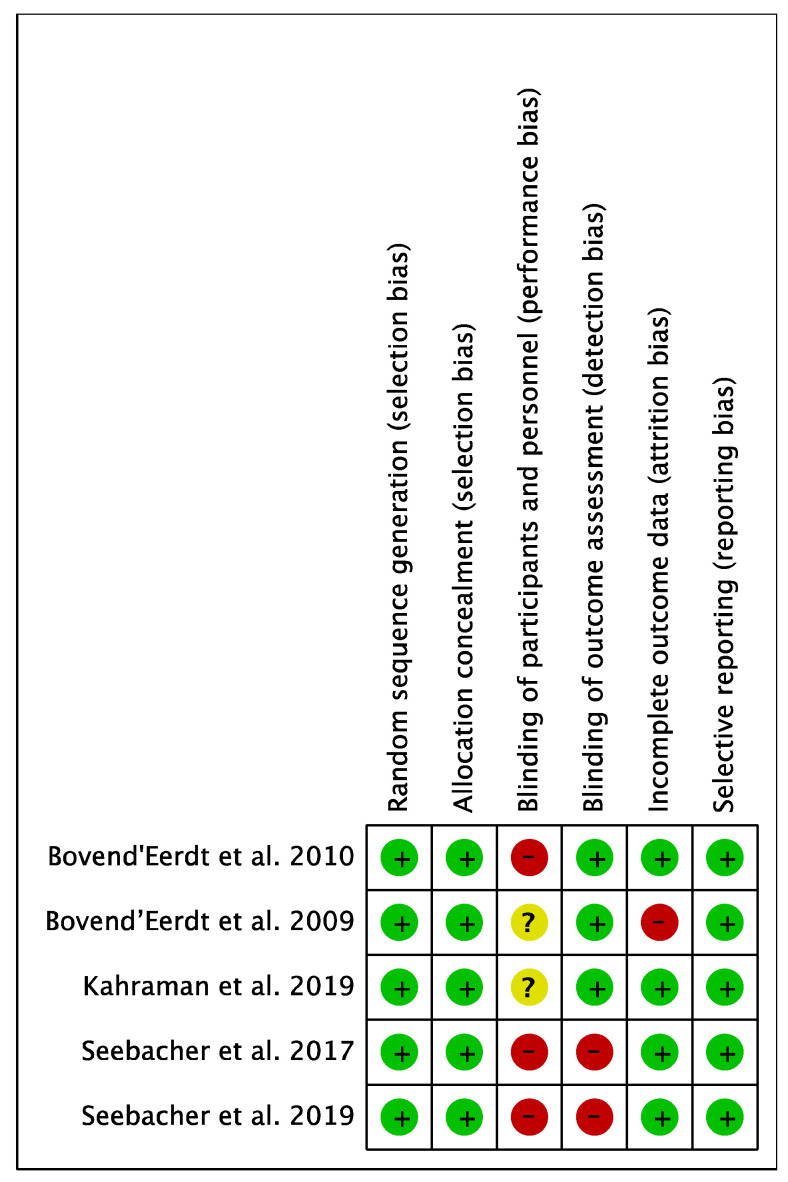
Risk of bias of the studies included in the systematic review.

**Figure 3:**
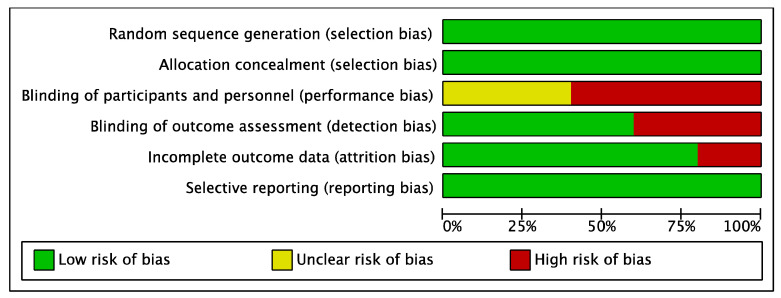
Overall risk of bias. The results are presented by percentages.

The mean methodological quality score was 17.4 points, with 12 [28] and 21 [21] points as the minimum and maximum obtained, respectively. Table 2 shows the results of the methodological quality of the included studies. Regarding the risk of bias of the studies, the two studies of Seebacher et al. [20,29] reached the highest risk of bias. Conversely, the study conducted by Kahraman et al. [21] presented the lowest risk of bias. Besides, in terms of the risk of bias among the studies, the lowest biases were obtained in the random sequence generation (selection bias), allocation concealment (selection bias) and the selective reporting (reporting bias). The highest value was found in the blinding of participants and personnel (performance bias). The results are shown in Figure 2 and Figure 3. 

### 3.2. Synthesis of Results

The most relevant characteristics of the studies included in this systematic review are shown in Table 3. 

### 3.3. Participant Characteristics

A total of 198 pwMS were studied. All samples were exclusively from pwMS except the articles published by Bovend’Eerdt et al. (2009) [28] and Bovend’Eerdt et al. (2010) [30], which included several types of patients with neurological pathologies, but only pwMS were considered. The average age of the individuals studied was between 35 and 52 years. Studies exclusively analysing pwMS did not exceed 3 points in the expanded disability status scale (EDSS) [31], ranging from 2 to 2.6 (mild disability in one function or very small disability in two functions). The EDSS is used to measure the degree of disability in pwMS and represents the most widely used tool to measure disease outcomes in clinical trials. It is a feasible scale for assessing the effectiveness of clinical interventions and to monitor disease progression [31].

The last two studies [28,30], which include other types of neurological patients, did not show EDSS scores, and only one [30] referred to a low degree of disability as a criterion for inclusion in the study.

The time since diagnosis in most studies varied from 4 to 19.7 years. When the chronicity of the pathology was not indicated, it was reported that at least over three months had elapsed since study participants had their last outbreak.

### 3.4. Intervention Characteristics

All studies used guided (involving any internal/external stimulus to provide feedback) MI, except Kahraman et al. [21] and Bovend’Eerdt et al. (2009) [26]. The most common guide was verbal, sometimes combined with music and a metronome. Furthermore, Holmes and Collins [32] used the physical, environment, task, timing, learning, emotion, perspective model (PETTLEP) approach. The PETTLEP model was developed to aid sports psychologists in designing and delivering MI interventions for athletes, and its application has been recommended for neurological patients [33]. 

The intervention duration was very heterogeneous, ranging from 17 to 30 min, the total number of sessions varied from 10 to 56, and the sessions per week from 4 to 8.

### 3.5. Outcomes Measures

Each of the variables is detailed below with the measurement instruments used for their evaluation.

#### 3.5.1. Gait

Four articles [20,21,29,30] proposed the use of MI to improve walking parameters in pwMS. These parameters were walking speed and distance, functionality and perceived walking ability. Despite the heterogeneity of the samples and methodologies, the majority of them showed significant improvements with the application in the MI intervention group compared to non-intervention.

The most studied parameter was walking speed, and the most used outcome measure to evaluate was Timed 25-Foot Walk (T25FW) used by three studies [20,21,29]; this test shows obvious real-world relevance, and has been strongly correlated with other measures of walking and lower limb function in pwMS [34]; The 6-Minute Walk Test (6MWT) was measured by two studies [20,29] and the 2-Minute Walk Test (2MWT) by Kahraman et al. [21]. Despite not being valid to determine aerobic capacity in PwMS, the validation of the 2MWT with other functional measures (like the TUG) has been demonstrated in pwMS; this test provides an efficient and practical alternative to the 6MWT [35]. In the case where different MI interventions were compared, verbally guided MI obtained the best results, as reported by Seebacher et al. [20]. Other variables, such as dynamic balance during walking and perceived walking ability were also significantly improved in the MI group in the study by Kahraman et al. [21].

Other gait parameters related to functionality were evaluated using the Rivermead Mobility Index (RMI) and Timed Up and Go (TUG) by the Bovend’Eerdt studies [28,30]. These results were less extrapolated since the number of pwMS was especially small. Kahraman et al. [21] used the 12-Item Multiple Sclerosis Walking Scale (MSWS-12) to measure the subjective impact of the disease on gait performance in these patients, obtaining significant improvements in the MI group.

#### 3.5.2. Balance

This variable was measured in the study by Kahraman et al. [21] using the limits of stability measurement and three tests: Activities-specific Balance Confidence (ABC), Postural Stability Test (PST) and the dynamic balance during gait through the Dynamic Gait Index (DGI). They found significant improvements after the use of MI, both in static and dynamic balance, as well as during the performance of fundamental tasks in their daily lives.

#### 3.5.3. Fatigue

The most often used scale to measure fatigue was the Modified Fatigue Impact Scale (MFIS), a specific fatigue scale in pwMS, which was used in three studies [20,21,29]. All the studies showed significant improvements in the MFIS using MI. Specifically, there were higher differences in the items referring to physical fatigue, followed by cognitive fatigue in the study by Seebacher et al. (2019) [20], compared to the other studies. Seebacher et al. (2017) [30] only obtained significant improvements on fatigue in the group receiving verbal guides and the metronome, compared to the musical guide and the control groups.

#### 3.5.4. Quality of Life

Three studies [20,21,29] measured QOL, and all the studies obtained significant results in favour of MI. The most used QOL scale was the Multiple Sclerosis Impact Scale-29 (MSIS-29), and it was used by the two studies of Seebacher et al. [20,29]. Another specific scale for pwMS was the Multiple Sclerosis International Quality of Life questionnaire (MUSI-QOL), as used by Kahraman et al. [21]. Other generic QOL scales that were used were the Short Form-36 Health Survey (SF-36) and the Euroquol-5D-3L Questionnaire (EQ-5D-3L).

#### 3.5.5. Secondary Outcomes

##### Upper Limb Function

Bovend’Eerdt et al. (2010) [30] used the Action Research Arm Test (ARAT) to measure the upper limb function. This scale evaluates the ability to handle and transport smaller and larger objects and is recommended as a quantification of arm function in pwMS, although it suffers from poor sensitivity to mild impairment [36]. No significant improvements were obtained after the intervention.

##### Muscular Tone and Range of Movement

Bovend’Eerdt et al. (2009) [28] used The Modified Ashworth Scale (MAS) and the ElectroGoniometer to measure the muscle response during passive stretching and range of movement. MAS scale is the most commonly used scale for assessing the degree of spasticity; however, the validity, reliability and sensitivity of this scale have been challenged [37]. The use of the ElectroGoniometer allows the evaluation of how this affects the range of movement. The results of this study showed no significant differences in EG and MAS measurements between the muscle relaxation and MI group.

##### Functional Independence

The two studies published by Bovend’Eerdt et al. [28,30] measured the impact of MI on functional independence. Bovend’Eerdt et al. (2010) [30] measured the functional impact of MI treatment on activities of daily living through the Nottingham Extended Activities of Daily Living (NEADL) scale and the Goal Attainment Scale (GAS). The NEADL seems to be appropriate for the assessment of disability in pwMS, but the range of items needs to be extended. The Goal Attainment Scale (GAS) is an individualised outcome measure, including goal selection and goal scaling, in order to calculate the patient goals achievement. Only significant post-intervention improvements in GAS were observed. Bovend’Eerdt et al. (2009) [28] used the Barthel Index (BI), which is a commonly used scale measuring functional independence, and it has been validated for pwMS [38]. No significant improvements were obtained after the intervention.

## 4. Discussion

The results of the present study focused on the main aspects of the MI technique for interventions on pwMS, its applicability, and the key features to be considered for implementing future trial protocols. One main aspect is the type of MI used, as it can be implemented with metronome, verbal, musical guides or following the PETTLEP [32] model. In this respect, the main interest of the PETTLEP model is on the influence of domains, such as the emotional one, on motor functioning. Most studies [20,21,29] used external guides in their protocols. In line with other authors [39], it has been shown that there are more relevant improvements with the use of these kinds of guides. Results showed that pwMS could better imagine movements when they are provided with external signals during MI intervention. In particular, Kharaman et al.’s [21] results support this, especially underscoring the positive effects of MI on balance, walking speed, fatigue and QOL.

The degree of disability, the chronicity of the disease and the age of the subjects also influence the performance of the technique. Cognitive and motor dysfunction may be associated with impaired motor imaging capabilities, which raises questions about the applicability and efficacy of this technique in neurological diseases and in pwMS. In this line, Heremans et al. [40] explained how cognitive dysfunction in pwMS affects the accuracy and duration of MI. In the same way, Tabrizi et al. [41] examined MI capabilities in pwMS with low scores on the EDSS and proved that there are significant differences compared to healthy controls. McInnes et al. [42] concluded that subjects with damage to specific brain structures, including the parietal and frontal lobes, show impaired MI ability. Mulder et al. [43] investigated the ability of different age groups for MI and found that elderly patients need an adaptation of the technique to their cognitive abilities. In particular, many of the subjects showed significant differences between first-person and third-person MI representations, with the former raising greater difficulties. Therefore, decisions about the use of MI in neurorehabilitation should be based, in part, on the specific functional profile of the patient’s underlying pathophysiology. 

Regarding the intensity of the therapy, while Seebacher et al. [20,29] used a more intense strategy in terms of the number of sessions and weeks of application, Kahraman et al. [21] reduced the intensity to 16 sessions an extended it for 8 weeks. Both studies showed the positive effects of MI, so it can be concluded that it is possible to reduce the number of MI sessions due to the influence of time on better processing of the MI-related changes. 

Telerehabilitation based on an MI [21] programme was introduced in 2019 as an innovative intervention applied to pwMS. Its results were favourable in improving motor and cognitive functions. Therefore, this study suggested that telerehabilitation combined with MI seemed feasible and applicable in pwMS, although the intervention should be individualised for each patient and situation by the therapist. 

Despite the lack of studies, partly due to the novelty of the technique in neurorehabilitation of pwMS, and due to the methodological differences between the few studies, it is difficult to obtain definitive conclusions on the effectiveness of MI in pwMS. However, considering the present systematic review results, qualitative analysis can be envisaged to deepen the different aspects of the considered variables, the technique and the application of MI in pwMS, which could guide future clinical trial protocols.

### 4.1. Gait

In addition to the positive results through the use of generic MI shown, the studies distinguished among the different types of MI application (music guided, music + verbal guidance, unguided or with the application of the PETTLEP model). Kahraman et al. [21] obtained less difference after the intervention, showing 0.33 s compared to the 0.8/0.9 s average of the studies by Seebacher et al. [20,29]. This discrepancy can be explained because patients with greater walking impairment showed a greater response to treatment or to the use of combined musical and verbal guides. Kahraman et al. [21] did not find alterations in the improvement of the 2-min walking test (2MWT) [35], in contrast with Seebacher et al. [20,29], who used the 6-min walking tests (6MWT). Although the results of these tests can be comparable [44], only the Seebacher et al. [20,29] findings could be considered as clinically significant [45]. Since rhythm-based music interventions can improve the gait parameters of velocity and cadence in pwMS [46], the results suggest that the combination of verbal and musical-guided MI should be used to improve the spatiotemporal parameters of gait.

### 4.2. Balance

Kahraman et al. [21] reported the static and dynamic balance variables using MI, showing significant differences in improving balance in pwMS. According to the literature [47,48], 50–60% of pwMS experienced falls during walking due to a loss of dynamic balance and differences in the biomechanical pattern [49]. The measurement of this variable using MI is clinically interesting, given its functional impact on pwMS. The instrumentalised evaluation of this variable in future studies will support the findings [50]. In the case of pwMS, visual disturbances can greatly influence balance. Although there is still no solid evidence on the supremacy of some sensory cues over others [51], it seems that visual cues are more important than auditory cues in balance in these patients [52]. However, these results suggest that the non-use of visual guidance, as in the case of the MI, did not prevent optimal results in improving balance through their use. The telerehabilitation modality used did not seem to affect the results either.

### 4.3. Fatigue

The significant results obtained in terms of fatigue, one of the most prevalent symptoms in pwMS, are encouraging. Cognitive mechanisms seem directly to interfere with fatigue, and the use of the MFIS is relevant because it takes into account both mental and physical fatigue [53,54], especially in a technique in which the cognitive demand may be greater than other physiotherapeutic techniques. Therefore, the duration of the sessions is also a factor to consider when carrying out an MI intervention due to the mental fatigue that pwMS often experience. Mental fatigue appears as an explanatory variable for cognitive performance and specifically in tasks that require speed of processing and executive control [55]. The length of the session in the studies included in this systematic review vary from 30 min [21] to 17 min [20,29], and all the protocol used contributed to the improvement of both mental and physical fatigue.

### 4.4. Quality of Life

The QOL is an important variable to take into account in physical therapy interventions and is key in the choice of techniques. Together with the scientific evidence of improvement in other functional variables, the results are very positive in terms of improvement in this area. It is recommended to include this in any future research in this field. Due to the deficits in motor control that pwMS present and which negatively affect QOL, the development of easy and convenient rehabilitation techniques is crucial in the clinical setting. Therefore, MI is a promising, cost-effective, and non-invasive complement to conventional therapy, which reduces deterioration and improves certain functional outcomes, with the possibility of carrying out this task at home [13,20].

### 4.5. Upper Limb Function

No significant improvements were obtained after intervention in the study of Bovend’Eerdt et al. [30], although the authors stated that the small sample size and intervention dose used could have affected the results. Furthermore, according to the authors, more support and training should be received by the therapists and patients in order to achieve the motor recovery of pwMS using the MI technique. Since the relationship between the upper and lower limb impairments in pwMS was only moderate [56], the implementation of studies analysing the effects of MI-based interventions that focus on the upper limb, such as the one described, are recommended in order to obtain a global overview of the overall motor function in pwMS.

### 4.6. Muscular Tone and Range of Movement

It should be considered that the sample size of the study of Bovend’Eerdt et al. (2009) [28] was the smallest (only a small number of the total were pwMS) and the protocol of action was not homogeneous, varying from 8 to 56 sessions and applied by caregivers or physical therapists. However, the authors commented that in the MI group, the feelings of discomfort during stretching were much lower, which is a positive aspect in clinical practice with patients with shortening and spasticity. With this regard, a measurement is needed that takes into account spasticity globally to make well-founded conclusions [57] and establish correlations between improvements in spasticity and its impact on balance and gait.

### 4.7. Functional Independence

Despite the non-significant results, which may be related to the aforementioned factors in the previous section, the authors used different scales that could help to establish a complete diagnosis through the Functional Classification of Independence [58]. It is essential to establish this diagnosis to develop objectives that allow the programming of interventions aimed at improving the activities of daily living of patients.

### 4.8. Study Limitations

Some limitations were found in this systematic review. One limitation was related to the use of strict inclusion criteria (only isolated MI interventions were included). Another limitation was the inclusion of studies analysing patients with different neurological disorders, the heterogeneity of the MI intervention protocols used by the studies, and the differences in disability levels. Future research should be based on controlled and randomised clinical trials, with patients exclusively with multiple sclerosis, with a proper and specific methodology that reinforce the use of MI for the improvement of variables as gait, fatigue, balance, and QOL. Furthermore, it would be appropriate to further investigate whether MI as a complementary treatment to conventional therapy would be beneficial to apply at the beginning of sessions, at the end of sessions or in stand-alone sessions. 

## 5. Conclusions

Although the evidence is limited, the use of MI through the combination of musical and verbal guides, compared to non-intervention or other interventions, can produce positive effects on walking speed and distance, fatigue and QOL in pwMS who have low EDSS scores. In addition, MI obtained significant improvements in dynamic balance and perceived walking ability. However, these results should be taken with caution because of the limited number and heterogeneity of the studies analysed.

Preliminary results suggested that interventions using the MI technique, applied with a minimum of 16 sessions, can be effective in the functional recovery of these patients. Nevertheless, due to the limited number of studies analysed, the results must be taken with caution. 

Despite the development of MI in the neurorehabilitation of patients with stroke and Parkinson’s disease being higher, it is necessary to continue researching on pwMS. The results obtained seem to be promising, but there are limitations when the technique is applied in the presence of cognitive impairments, being easier to use in patients with low EDSS scores. In this way, we encourage authors to use large sample sizes to analyse the effects of MI interventions on different clusters based on EDSS, with specific interventions for both upper and lower limbs.

In conclusion, the number of clinical trials with high methodological quality using MI-based interventions for motor recovery of pwMS is low. Future research needs to be based on randomised controlled trials with homogeneous intervention programmes and disability levels, employing measuring instruments assessing pain, spasticity, risk of falls, QOL and functional scales. These improvements will allow obtaining more conclusive findings of the real impact of the technique on pwMS lives. 

## Figures and Tables

**Table 1:** Search strategy for the different databases.

Databases	Search
Medline/PubMed, Scopus, CINAHL, Cochrane, Web of Science	(“motor imagery” OR “mental imagery” OR “mental practice”) AND “multiple sclerosis” AND rehabilitation
PEDro	Motor imagery in multiple sclerosis

**Table 2:** Methodological quality of the studies included in the systematic review.

	Kahraman et al. 2019 [21]	Seebacher et al. 2019 [20]	Seebacher et al. 2017 [29]	Bovend’Eerdt et al. 2010 [30]	Bovend’Eerdt et al. 2009 [28]
1. Study quality					
Hypothesis/aim	1	1	1	1	1
Outcomes	1	1	1	1	1
Eligibility criteria	1	1	1	1	1
Interventions	1	1	1	1	1
Confounders	0	0	0	0	0
Findings	1	1	1	1	1
Random variability	1	1	1	1	1
Adverse events	1	0	0	1	1
Lost to follow-up	1	1	1	1	1
Probability values	1	1	1	1	1
2. External validity (study bias)					
Source population	0	0	0	0	0
Illustrative sample	0	0	0	0	0
Illustrative treat	1	0	0	0	0
3. Internal validity (study bias)					
Blinding of subjects	1	0	0	0	0
Blinding	1	1	1	1	0
“Data dredging”	1	1	1	1	1
Follow-up adjusts	1	1	1	1	1
Statistical tests	1	1	1	1	1
Compliance	1	1	1	0	1
Outcomes	1	1	1	0	1
4. Internal validity (confounding and selection bias)					
Source of patients	1	0	1	0	0
Recruitment period	1	1	1	1	1
Randomization	1	1	1	0	0
Concealment	0	1	1	0	0
Analysis	0	0	0	0	0
Loss to follow-up	1	0	0	0	0
5. Power					
Effect	1	0	0	1	0
TOTAL SCORE	21	19	19	14	12
PORCENTAGE (%) *	61	59	59	42	38

* Percentage of the maximum score (32 points).

**Table 3:** Synthesis of Results.

Authors (Year)	Sample	Type of MI	Age (Average)	Stadium	N Sessions, Temporality	Performance of Measurement	Results
Kahraman et al. 2019 [21]	N = 33 TRBM = 19 C = 14	No guided Tele-MI, PETTLEP	35.2 (38.3–42.7)	(average EDSS = 2) Chronicity 4 years.	16 sessions (30 min), 0/1 session/day, 2 days/week, 8 weeks	T25FW, TUG, 2MWT, MSWS-12, DGI, ABC, LOS, PST, MFIS, MUSIQOL	In the intervention group, significant improvements in walking speed (*p* = 0.007), perceived walking ability (*p* = 0.008), dynamic balance (*p* = 0.002), fatigue (*p* = 0.001) and quality of life (*p* = 0.002)
Seebacher et al. 2019 [20]	N = 59 MVMI = 19 MMI = 20 MING = 20	Guided by music and verbal and no guided	44.36 (39.5–49.5)	(EDSS 2.6) Chronicity + 3 months	24 sessions (17 min), 0/1 session/day, 6 days/week, 4 weeks	T25FW, 6MWT, MFIS, MSIS-29	Within-group comparisons showed that all three interventions significantly improved walking speed and walking distance. Between-group analyses show significant improvements, in MVMI group, in distance (*p* = 0.024) and walking speed (*p* = 0.001), in fatigue (*p* = 0.030) and quality of life (*p* = 0.024)
Seebacher et al. 2017 [29]	N= 101 MVMI = 34 MTVMI = 34 C = 33	Guided by verbal, music, or metronome	44.1 (39.6–46.3)	(EDSS 2) Chronicity + de 3 months	24 sessions (17 min), 0/1 session/day, 6 days/week, 4 weeks	T25FW, 6MWT, MSWS-12, MFIS, MSIS-29, SF36, EQ-5D-3L	Significant improvements in walking speed (*p* < 0.0001) and distance (*p* < 0.0001) (within-group comparisons). Physical fatigue (*p* = 0.001) (only significant in the MVMI group) quality of life (only significant in MVMI group) (*p* = 0.005)
Bovend’Eerdt et al. 2010 [30]	N = 30 C = 0 MS MIG = 1 MS	Guided by verbal	51.2 ± 11	EDSS low Chronicity 10 years	10 sessions, 3 days/week, 3 weeks 2 days/week, 2 weeks	TUG, RMI, ARAT, NEAD, GAS	Poor adherence to the treatment. Significant improvements post-intervention only in GAS (*p* < 0.001) but not over time (*p* = 0.845)
Bovend’Eerdt et al. 2009 [28]	N = 11(4 MS) PMR = 1 MS MI = 3 MS	No guided	48.25 ± 10	EDSS not detailed Chronicity 19 years.	8–56 sessions, 8 weeks	RMI, MAS, EG, BI	No significant differences were found between groups after intervention (*p* > 0.05).

(ABC) Activities-specific Balance Confidence; (ARAT) Action Research Arm Test; (BI) Barthel Index; (C) Control: no intervention; (DGI) Dynamic Gait Index; (EDSS) Expanded Disability Status Scale; (EG) Electrogoniometer; (EQ-5D-3L) Euroquol-5D-3L Questionnaire; (GAS) Goal Attainment scale; (LOS) Limits of Stability; (MAS) Modified Ashworth Scale; (MFIS) Modified fatigue impact scale; (MI) Motor imagery; (MIG) Motor Imagery Group; (MING) Motor imagery no guided; (MMI) Music motor imagery; (MS) Multiple Sclerosis; (MSIS-29) Multiple Sclerosis Impact Scale-29; (MSWS-12) 12-Item Multiple Sclerosis Walking Scale; (MTVMI) Metronome verbally motor imagery; (MusiQoL) Multiple Sclerosis International Quality of Life questionnaire; (MVMI) Music verbally motor imagery; (N) Total number of patients; (NEADL) Nottingham Extended Activities of Daily Living; (PASAT) Paced Auditory Serial Addition Test; ((PETTLEP) Physical, Environment, Task, Timing, Learning, Emotion, Perspective model; (PMR) Progressive muscle relaxation; (PST) Postural Stability Test; (RMI) Rivermead Mobility Index; (SF-36) Short Form-36 Health Survey; (TUG) Timed Up and Go; (TRBMI) Telerehabilitation-bases motor imagery; (T25FW) Timed 25-Foot Walk; (2MWT) 2-Minute Walk Test.

## Data Availability

No new data were created in this study. Data sharing is not applicable to this article.

## Supplementary Materials

The following are available online at https://www.mdpi.com/1660-4601/18/2/498/s1, Table S1: Search strategy for PubMed database. List S1: Excluded articles and reasons.
