# Supplementary material for: Effectiveness of Motor Imagery on Motor Recovery in Patients with Multiple Sclerosis: Systematic Review
Source: Int J Environ Res Public Health. 2021 Jan 9;18(2):498. doi: 10.3390/ijerph18020498 (PMC7827037; doi:10.3390/ijerph18020498)
Supplement: Supplementary file 1 [file ijerph-18-00498-s001.pdf]

Supplementary materials:

Table S1: Search strategy for PubMed database.

**Complete search strategy**	
**#1**	Search ("Multiple sclerosis" [Mesh])
**#2**	Search ("motor imagery" OR "mental imagery" OR "mental practice")
**#3**	Search ("Rehabilitation" [Mesh])
**#4**	Search (#1 AND #2 AND #3)

No filters were used according to the study type.

List S1: Excluded articles and reasons.

RECORDS EXCLUDED BASED ON EXCLUSION CRITERIA (N = 16)

Not related to targeted outcomes (n = 11)

1. Tacchino A, Saiote C, Brichetto G, Bommarito G, Roccatagliata L, Cordano C, Battaglia MA, Mancardi GL, Inglese M. Motor Imagery as a Function of Disease Severity in Multiple Sclerosis: An fMRI Study. Front Hum Neurosci. 2018;11:628. doi: 10.3389/fnhum.2017.00628.
2. Tabrizi YM, Mazhari S, Nazari MA, Zangiabadi N, Sheibani V. Abnormalities of motor imagery and relationship with depressive symptoms in mildly disabling relapsing-remitting multiple sclerosis. J Neurol Phys Ther. 2014;38(2):111-8. doi: 10.1097/NPT.0000000000000033.
3. Tabrizi YM, Zangiabadi N, Mazhari S, Zolala F. The reliability and validity study of the Kinesthetic and Visual Imagery Questionnaire in individuals with multiple sclerosis. Braz J Phys Ther. 2013;17(6):588-92. doi: 10.1590/S1413-35552012005000124.
4. Wondrusch C, Schuster-Amft C. A standardized motor imagery introduction program (MIIP) for neuro-rehabilitation: development and evaluation. Front Hum Neurosci. 2013;7:477. doi: 10.3389/fnhum.2013.00477.
5. Heremans E, D'hooge AM, De Bondt S, Helsen W, Feys P. The relation between cognitive and motor dysfunction and motor imagery ability in patients with multiple sclerosis. Mult Scler. 2012;18(9):1303-9. doi: 10.1177/1352458512437812.
6. Schuster C, Lussi A, Wirth B, Ettlin T. Two assessments to evaluate imagery ability: translation, test-retest reliability and concurrent validity of the German KVIQ and Imaprax. BMC Med Res Methodol. 2012;12:127. doi: 10.1186/1471-2288-12-127.
7. Heremans E, Nieuwboer A, Spildooren J, De Bondt S, D'hooge AM, Helsen W, Feys P. Cued motor imagery in patients with multiple sclerosis. Neuroscience. 2012;206:115-21. doi: 10.1016/j.neuroscience.2011.12.060.
8. Heremans E, Vercruysse S, Spildooren J, Feys P, Helsen WF, Nieuwboer A. Evaluation of motor imagery ability in neurological patients: a review. Mov Sport Sci/Sci Mot. 2013;82:31-38. DOI: https://doi.org/10.1051/sm/2013097
9. Kahraman T, Savci S, Ozdogar AT, Gedik Z, Idiman E. Effects of anxiety on motor imagery ability in patients with multiple sclerosis. Türk Fizyoterapi ve Rehabilitasyon Dergisi. 2018;29(1): 19-26.
10. Maguire BL. The effects of imagery on attitudes and moods in multiple sclerosis patients. Altern Ther Health Med. 1996;2(5):75-9.
11. Ernst A, Sourty M, Roquet D, Noblet V, Gounot D, Blanc F, de Seze J, Manning L. Benefits from an autobiographical memory facilitation programme in relapsing-remitting multiple sclerosis patients: a clinical and neuroimaging study. Neuropsychol Rehabil. 2018;28(7):1110-1130. doi: 10.1080/09602011.2016.1240697.

Not patients with Multiple Sclerosis (n = 1)

1. Kawakami M, Okuyama K, Takahashi Y, Hiramoto M, Nishimura A, Ushiba J, Fujiwara T, Liu M. Change in Reciprocal Inhibition of the Forearm with Motor Imagery among Patients with Chronic Stroke. Neural Plast. 2018;2018:3946367. doi: 10.1155/2018/3946367.

Not isolated Motor Imagery (n = 3)

1. Pawlukowska W, Dobrowolska N, Szylinska A, Koziarska D, Meller A, Rotter I, Nowacki P. Influence of RehaCom Therapy on the Improvement of Manual Skills in Multiple Sclerosis Subjects. Ann Rehabil Med. 2020;44(2):142-150. doi: 10.5535/arm.2020.44.2.142
2. Behrendt F, Schuster-Amft C. Using an interactive virtual environment to integrate a digital Action Research Arm Test, motor imagery and action observation to assess and improve upper limb motor function in patients with neuromuscular impairments: a usability and feasibility study protocol. BMJ Open. 2018;8(7):e019646. doi: 10.1136/bmjopen-2017-019646.
3. Leonard G, Lapierre Y, Chen JK, Wardini R, Crane J, Ptito A. Noninvasive tongue stimulation combined with intensive cognitive and physical rehabilitation induces neuroplastic changes in patients with multiple sclerosis: A multimodal neuroimaging study. Mult Scler J Exp Transl Clin. 2017;3(1):2055217317690561. doi: 10.1177/2055217317690561.

Not Motor Imagery intervention (n = 1)

1. Kaur D, Kaur K, Billore N, Kumar G, Kumar Singh A. Mental Tai-Chi based exercise programme vs Tai-Chi for Indian Multiple Sclerosis patients: A pilot study. International Journal of current research and review. 2014;6(19):24-30.

FULL-TEXT ARTICLES EXCLUDED (N = 17)

Not clinical trials or pilot studies (n = 16)

1. Soler B, Ramari C, Valet M, Dalgas U, Feys P. Clinical assessment, management, and rehabilitation of walking impairment in MS: an expert review. Expert Rev Neurother. 2020;20(8):875-886. doi: 10.1080/14737175.2020.1801425.
2. Kaur J, Ghosh S, Sahani AK, Sinha JK. Mental imagery training for treatment of central neuropathic pain: a narrative review. Acta Neurol Belg. 2019;119(2):175-186. doi: 10.1007/s13760-019-01139-x.
3. Hanson M, Concialdi M. Motor imagery in multiple sclerosis: exploring applications in therapeutic treatment. J Neurophysiol. 2019;121(2):347-349. doi: 10.1152/jn.00291.2018.
4. Ghai S, Ghai I. Effects of Rhythmic Auditory Cueing in Gait Rehabilitation for Multiple Sclerosis: A Mini Systematic Review and Meta-Analysis. Front Neurol. 2018;9:386. doi: 10.3389/fneur.2018.00386.
5. Castelnuovo G, Giusti EM, Manzoni GM, Saviola D, Gatti A, Gabrielli S, Lacerenza M, Pietrabissa G, Cattivelli R, Spatola CA, Corti S, Novelli M, Villa V, Cottini A, Lai C, Pagnini F, Castelli L, Tavola M, Torta R, Arreghini M, Zanini L, Brunani A, Capodaglio P, D'Aniello GE, Scarpina F, Brioschi A, Priano L, Mauro A, Riva G, Repetto C, Regalia C, Molinari E, Notaro P, Paolucci S, Sandrini G, Simpson SG, Wiederhold B, Tamburin S. Psychological Treatments and Psychotherapies in the Neurorehabilitation of Pain: Evidences and Recommendations from the Italian Consensus Conference on Pain in Neurorehabilitation. Front Psychol. 2016;7:115. doi: 10.3389/fpsyg.2016.00115.
6. Braun S, Kleynen M, van Heel T, Kruithof N, Wade D, Beurskens A. The effects of mental practice in neurological rehabilitation; a systematic review and meta-analysis. Front Hum Neurosci. 2013;7:390. doi: 10.3389/fnhum.2013.00390.
7. Slifkin A, Bethoux F, Stough D, Charlotte M, Bialko C, Eder J. Mental practice of action and rehabilitation of multiple sclerosis. 2007. Poster presented at the 12th Annual ACTRIMS Meeting, Washington, DC.

8. Motaharinezhad F, Seyed S, Zeinali R. The impact of mental practice on sleep quality, fatigue, functional balance and gaiting in patitent with multiple sclerosis: A case report. Koomesh. 2016;17(4):944-949.
9. Christakou A. Motor control and motor learning: Rehabilitation instruments and techniches. Epitheorese klinikes Farmakologias kai Farmakokinetikes. 2013;31(3):234-242.
10. Bovend'Eerdt TJ. Motor Imagery Practice in Neurological Rehabilitation. Clinical trial protocol. ClinicalTrials.gov Identifier: NCT00618085 https://clinicaltrials.gov/show/NCT00618085
11. Kahraman T. Motor Imagery Training in Persons With Multiple Sclerosis. ClinicalTrials.gov Identifier: NCT02781142 https://clinicaltrials.gov/show/NCT02781142
12. Azin M. Study of transcranial magnetic stimulation effect on motor imagery rehabilitation and movement improvement in multiple sclerosis. Iranian Registry of Clinical Trials. IRCT registration number: **IRCT2013042313100N1**
13. Weigel M, Fleming R, Hutchinson B, Magee WL, Su W. (REH25) Orchestrating a New Path for Multiple Sclerosis Rehabilitation: Empowering Patients Through Both Physical and Music Therapies. International Journal of MS Care. 2020;22(S2):75.
14. Seebacher B, Kuisma R, Glynn A, Berger T. Exploring cued and non-cued motor imagery interventions in people with multiple sclerosis: a randomised feasibility trial and reliability study. Arch Physiother. 2018;8:6. doi: 10.1186/s40945-018-0045-0.
15. Seebacher B, Kuisma R, Glynn A, Berger T. Rhythmic cued motor imagery and walking in people with multiple sclerosis: a randomised controlled feasibility study. Pilot Feasibility Stud. 2015 Jul 11;1:25. doi: 10.1186/s40814-015-0021-3.
16. Catalan M, De Michiel A, Bratina A, Mezzarobba S, Pellegrini L, Marcovich R, et al. Treatment of fatigue in multiple sclerosis patients: a neurocognitive approach. Rehabil Res Pract. 2011 Sep;2011:670537.

Other language (n = 1)

1. Kharestani M, Abdar ME, Gholipour M. Changes Related to Performing Attentional Focus Imagery on Dynamic Balance in Patients with Multiple Sclerosis. Journal of Advances in Medical and Biomedical Research. 2017;25(108):96-106.
